# Increased body mass index is associated with specific regional alterations in brain structure

**DOI:** 10.1038/ijo.2016.42

**Published:** 2016-04-19

**Authors:** N Medic, H Ziauddeen, K D Ersche, I S Farooqi, E T Bullmore, P J Nathan, L Ronan, P C Fletcher

**Affiliations:** 1 Department of Psychiatry, University of Cambridge, Cambridge, UK; 2 Wellcome Trust-MRC Institute of Metabolic Science, University of Cambridge, Cambridge, UK; 3 Cambridgeshire and Peterborough NHS Foundation Trust, University of Cambridge, Cambridge, UK; 4 Medicines Discovery and Development, GlaxoSmithKline, Clinical Unit Cambridge, Cambridge, UK; 5 School of Psychological Sciences, Monash University, Melbourne, Victoria, Australia

## Abstract

**Background:**

Although obesity is associated with structural changes in brain grey matter, findings have been inconsistent and the precise nature of these changes is unclear. Inconsistencies may partly be due to the use of different volumetric morphometry methods, and the inclusion of participants with comorbidities that exert independent effects on brain structure. The latter concern is particularly critical when sample sizes are modest. The purpose of the current study was to examine the relationship between cortical grey matter and body mass index (BMI), in healthy participants, excluding confounding comorbidities and using a large sample size.

**Subjects:**

A total of 202 self-reported healthy volunteers were studied using surface-based morphometry, which permits the measurement of cortical thickness, surface area and cortical folding, independent of each other.

**Results:**

Although increasing BMI was not associated with global cortical changes, a more precise, region-based analysis revealed significant thinning of the cortex in two areas: left lateral occipital cortex (LOC) and right ventromedial prefrontal cortex (vmPFC). An analogous region-based analysis failed to find an association between BMI and regional surface area or folding. Participants' age was also found to be negatively associated with cortical thickness of several brain regions; however, there was no overlap between the age- and BMI-related effects on cortical thinning.

**Conclusions:**

Our data suggest that the key effect of increasing BMI on cortical grey matter is a focal thinning in the left LOC and right vmPFC. Consistent implications of the latter region in reward valuation, and goal control of decision and action suggest a possible shift in these processes with increasing BMI.

## Introduction

At present, >1.3 billion people across the world are overweight and an additional 600 million are obese. Overweight and obesity are the leading risk factors for deaths globally, being associated with increased incidence of type 2 diabetes, cardiovascular disease and some cancers.^1^Although these illnesses are known to have effects on the brain, there is now an increasing evidence that obesity itself is associated with altered brain structure, possibly mediated by physiological dysregulations, such as low-grade inflammation,^2^ which has been linked to brain atrophy.^3^ The relevance of obesity to brain structure and function is further emphasised by the finding that it is a risk factor for Alzheimer's disease in the elderly,^4^ and in younger adults and children, it is associated with poorer memory and executive function (reviewed in Smith *et al.*^5^).

A number of studies have assessed the relationship between body weight and brain structure, predominantly focusing on the grey matter volume (GMV; Supplementary Table 1). Strikingly, the findings have proven highly inconsistent whether one considers global or regional changes in GMV. Although several studies have reported decreased total GMV in obesity,^6, 7, 8, 9^ others have reported no reductions.^10, 11^ Although the majority of studies report decreases in grey matter in a number of regions,^6, 8, 12, 13, 14^ the precise localisation has varied widely and some studies have even reported increases in regional GMV in obese subjects.^8, 9, 14, 15, 16^ Such inconsistencies pose a major challenge, impeding progress in developing an understanding of the relationship between weight dysregulations and the brain.

There are a number of possible reasons for the inconsistencies. First, obesity is driven by complex environmental, behavioural, metabolic and genetic determinants. The obese phenotype as defined by body mass index (BMI) is a heterogeneous one, being the outcome of several possible causal paths. This is a limitation of the BMI-based phenotype not unique to this particular question, but such heterogeneity can lead to significant variability of findings, particularly when sample sizes are small. Second, as the aforementioned comorbidities have been independently linked to brain atrophy, they represent potential confounders whose effects are difficult to quantify and control for (for example, the effect of diabetic status on brain structure). The presence of such confounders can also lead to inconsistent findings, particularly if they are treated differently across studies.

The third reason relates to the methods used to examine brain structure. Different measures of morphometry are differentially sensitive and reflect different structural changes. Most studies so far have used voxel-based morphometry that produces a probabilistic measure of GMV. This measure is determined through a pipeline of magnetic resonance image transformation and warping without regard for anatomical features of the cortex. Moreover, it is a composite of cortical thickness and surface area, which though correlated, represent distinct biological aspects of the cortex, and have different genetic components.^17^ Here we sought to overcome this limitation using a surface-based morphometric method, allowing us to disambiguate these measures, and thus permit more precise biological interpretation.^18^

In short, despite the growing body of work demonstrating grey matter changes associated with obesity, the precise nature of these changes is obscured by a number of factors, including heterogeneity and varying methodologies. The purpose of the current study was to obviate some of these problems. To do this, we used surface-based morphometry to derive distinct measures of cortical grey matter structure, including cortical thickness, surface area and cortical folding, and explored their relationship with BMI in a large sample of young to middle-aged adults with no reported clinical morbidities.

## Materials and methods

### Participants

T1 magnetic resonance imaging (MRI) scans from 203 subjects were analysed (age *M*=32.3, s.d.=7.7, range 18–50; 79 females). All subjects were scanned in the Wolfson Brain Imaging Centre, as part of studies conducted by the Department of Psychiatry. Recruitment and MRI scanning of participants were approved by the National Health Service Local Research and University of Cambridge Psychology Research Committees. The scanning was conducted in accordance with the principles of the Declaration of Helsinki. All participants provided written informed consent.

All subjects were self-reported healthy, had no significant medical history, were not receiving any pharmacological treatment and reported no contraindications to MRI scanning. Ninteen subjects were smokers. BMI (weight in kilograms divided by the square of height in metres) was calculated for all participants from height and weight measures collected during the primary studies. The BMI range across the sample was 18.5–46.4 kg m^−2^ (*M*=28.5 kg m^−2^, s.d.=6.3 kg m^−2^).

### MRI acquisition

T1 MRI scans were acquired on two 3-T scanners, the Siemens Trio and Siemens Verio (Siemens Medical Systems, Erlangen, Germany). Magnetic resonance images were acquired with a sagittal MPRAGE T1-weighted, three-dimensional, inversion recovery gradient echo sequence with the following parameters: inversion time=900 ms; echo time=2.98 ms; repetition time=2300 ms; flip angle=9° voxel dimensions=1 × 1 × 1 mm; and acquisition time=9.14 min.

### MRI processing

Cortical reconstructions were generated using FreeSurfer,^19^ which was specifically developed for this purpose. The raw image data voxels were sub-sampled to voxels of size 1 mm^3^, following which the data were normalised for intensity. Radio-frequency bias field inhomogenieties were modelled and removed, followed by skull-stripping. The cerebral white matter was identified and the hemispheres were separated, tessellated and deformed to produce an accurate and smooth representation of the white–grey matter interface. Where inaccuracies occurred, reconstructions were edited manually. Surfaces were averaged across participants using a nonrigid high-dimensional spherical averaging method to align cortical folding patterns. This provides accurate matching of morphologically homologous cortical locations across subjects on the basis of each individual's anatomy while minimising metric distortion. The target used for registration was the white–grey matter surface boundary. At each vertex, cerebral cortex thickness was quantified as the shortest distance between the white–grey matter surface and the grey matter–cerebrospinal fluid surface. Continuous maps of surface area of the white–grey matter boundary were obtained by computing the surface area of each vertex in the standardised, spherical atlas space, mapped to each subject's native space. This provides a vertex-wise estimate of the relative surface area expansion or contraction of each location in atlas space. Cortical folding, or the degree of gyrification, was explored by computing the local gyrification index (LGI). In a vertex-wise manner, the LGI measures the ratio of cortex buried within sulcal folds and visible cortex on the outer surface. Cortical thickness and surface area maps were smoothed using a full width at half maximum 15-mm kernel, whereas LGI, due to its intrinsic smoothness, was smoothed with a smaller, full width at half maximum 5-mm kernel. For each subject, the global measures of total surface area, and average cortical thickness and LGI were also computed.

### Statistical analyses

First, we analysed the association between BMI and global measures of average cortical thickness, total surface area and average LGI. This was examined using mixed effects modelling in R,^20^ with a random effect for individual subjects. A Bonferroni correction was adopted to account for the measurement of three variables, leading to a corrected threshold of *P*<0.017. Second, we examined the association between BMI and cortical thickness, surface area and LGI at each vertex of the cortex. This was performed using general linear modelling implemented in FreeSurfer. Correction for multiple comparisons was performed at the cluster level using Monte Carlo simulation (10 000 repetitions), *P*_MC_<0.05, in addition to a Bonferroni correction to account for the analysis of three measures over two hemispheres.

Age and gender were controlled for in all models, by including them as covariates in the models. Given that scanning was performed on two different 3-T scanners (using the same scanning sequence), in all statistical models, scanner type was included as a covariate. In addition, in the global and vertex-wise analyses of cortical thickness and LGI, and vertex-wise analysis of surface area, global surface area was used a covariate. As FreeSurfer produces separate outputs for each hemisphere for the above global measures, hemisphere was also included as a covariate into these models.

## Results

One subject had an unusually thin cortex (<2mm). This subject was excluded as an outlier, which left 202 subjects for the analyses.

### Analyses of global measures

There was no association between BMI and global measures of average cortical thickness, total surface area or average LGI (Table 1). Although the age and BMI of subjects were moderately correlated (*r*=0.31, *P*<0.0001), no multicolinearity was detected in the estimated models (the variance inflation factors of all the predictors in the models were <2).

### Vertex-wise analyses

**Figure 1:**
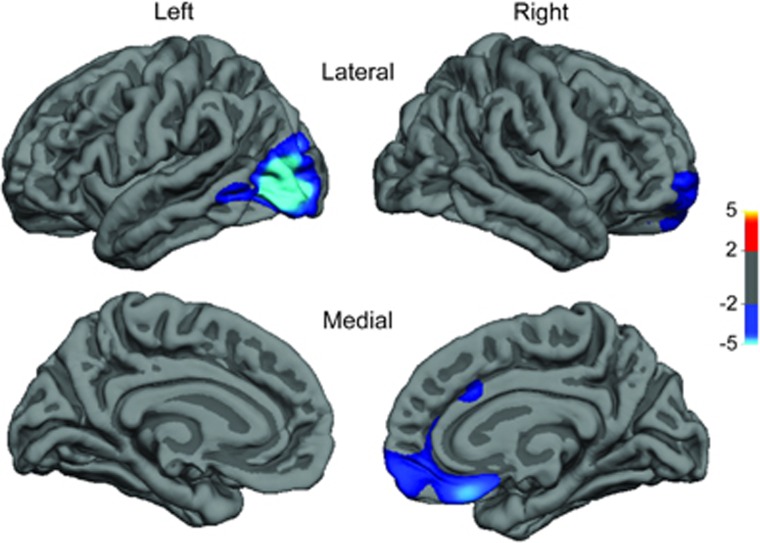
Lateral and medial view of the two clusters (right vmPFC and left LOC) whose cortical thickness exhibited a negative correlation with BMI. The colour bar represents the logarithmic scale of Monte Carlo cluster-wise corrected *P*-values (−log(10)*P*_MC_). Red indicates positive and blue indicates negative association.

**Figure 2:**
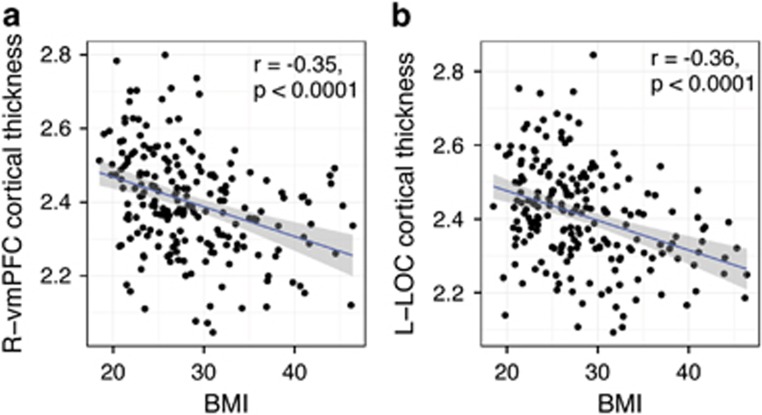
Scatter plots demonstrating the association between BMI and averaged cortical thickness in two clusters: right vmPFC (**a**) and left LOC (**b**).

Although BMI was not associated with surface area or LGI on the vertex-wise level, it was negatively correlated with cortical thickness in two clusters. The first cluster was located in the right ventromedial prefrontal cortex (vmPFC; Talairach peak: 5, 26, −21; *P*_MC_=0.0001), expanding to the frontopolar and anterior cingulate. The second cluster was located in the left lateral occipital cortex (LOC; Talairach peak: −41, −86, −1; *P*_MC_=0.0001; Figure 1; Table 2). The percentage of variance of cortical thickness explained by BMI in the right vmPFC and left LOC was 12.25% and 12.96%, respectively. Scatter plots demonstrating the association of cortical thickness and BMI in these two clusters are shown in Figure 2. *Post hoc* contrasts revealed that lean (BMI<25) and overweight subjects (25⩽BMI<30) did not significantly differ in the thickness of the right vmPFC or left LOC (*P*=0.29 and *P*=0.34, respectively), while obese subjects (BMI⩾30) had significantly thinner cortices in these clusters, compared with both lean (*P*<0.0001 in both clusters) and overweight subjects (*P*<0.0001 in both clusters). Including smoking status as a covariate into the above models (global and vertex–wise) did not change the presented results.

We explored whether there was overlap between the age-related and BMI-related effects on cortical thickness. The vertex-wise analysis of the association of cortical thickness with age, controlling for gender, scanner, global surface area and BMI revealed 11 distinct clusters in which age was negatively associated with cortical thickness (*P*_MC_<0.05, 10 000 repetitions: Supplementary Figure 1; Supplementary Table 2). Furthermore, including the interaction term in the model revealed that there was no region in the brain with an age-by-BMI interaction on cortical thickness (*P*_MC_<0.05, 10 000 repetitions), suggesting that the detected BMI-related thinning was separate from the age-related effects on thickness. Given the existing reports of gender differences in the association of obesity with brain structural changes (Supplementary Table 1), we also explored whether the established effect of BMI on cortical thickness could have been driven by the effects specific to men or women; however, there was no region in the brain with a gender-by-BMI interaction on cortical thickness (*P*_MC_<0.05, 10 000 repetitions).

## Discussion

This study was carried out in the context of a growing, but highly inconsistent literature exploring the impact of adiposity on the brain's grey matter structure. We optimised our approach by selecting a large set of structural images from adults who had no known clinical morbidity, and using a surface-based morphometric approach that permits the extraction of key parameters of cortical structure separately. We demonstrate in this large group of adults that there is no significant relationship between BMI, a well-established measure of adiposity, and global measures of cortical thickness, surface area or cortical folding. Using a spatially more precise, regionally specific approach, while there was no association between BMI and surface area or cortical folding, there was a robust reduction in cortical thickness associated with increasing BMI in two brain regions—the right vmPFC and left LOC. As age and global cortical surface area were controlled for, this is very unlikely to be an age-related or mechanical effect. Given that our volunteers reported no existing clinical condition increases our confidence that this specific alteration in cortical thickness is related to BMI, or BMI-associated subclinical metabolic dysregulations, rather than to clinical comorbidities of obesity.

There is growing evidence that cortical thickness has a systematic and interpretable relationship to underlying cytoarchitecture. Gradients in cortical thickness across the brain have been shown to reflect the brain's functional hierarchies.^18^ Thinner cortex is thought to have higher neuronal density and shorter axonal/dendritic processes.^21^ The BMI-associated cortical thinning in the vmPFC and LOC therefore might be linked to greater neuronal density and reduced dendritic arborisation of these regions in obese individuals. Given that the thinning was observed over and above the effects of age and total surface area (in addition to age-related thinning, larger brains tend to have thinner cortices), we speculate that it reflects more fundamental changes in cytoarchitecture. Such changes have been linked to function: in the most extreme examples, changes in dendritic arborisation and spine structure are commonly observed in brain tissue of patients with various types of intellectual disabilities.^22^ Mutations affecting synaptic morphology and plasticity have been implicated in intellectual disability, schizophrenia and autism spectrum disorders.^23, 24^ Furthermore, morphometric studies have linked variations in cortical thickness across normal populations to a number of cognitive attributes, including intelligence,^25^ executive function^26^ and attention.^27^ In short, cortical thickness s a parameter that relates to brain cytoarchitecture and function. A key question here is whether any conclusions can be drawn from the localisation of BMI-related changes observed in this sample and we begin by considering the known functions of these regions.

The vmPFC has a well-established role in decision-making, and particularly, in concert with the dorsolateral prefrontal cortex, in the control of goal-directed behaviour.^28^ Further, in a food-related task, De Wit^29^ demonstrated that the transition from goal-directed to habit-based responding is associated with an attenuation in responses in this region. We might therefore speculate that reduced thickness of the vmPFC is associated with a shift in balance between goal-directed and habitual or stimulus-dependent responding. Such a shift would account for an enhanced tendency to consume in response to the array of environmental cues that populate our environment and are continually driving us towards consumption, a drive that might otherwise be resisted by adherence to long-term goals and plans. It is noteworthy that, compared with lean subjects, poorer performance on the Iowa gambling task (originally developed and used by Bechara *et al.*^30^ in characterising decision-making deficits in vmPFC-lesioned patients) has been reported in obese subjects by multiple studies (reviewed in Vainik *et al.*^31^). Similarly, overeating and obesity have been linked to increased impulsivity—a personality trait operationalised through reduced response inhibition and/or steeper delay discounting.^32^ Studies of executive function, comprising various processes underpinned by goal-directed control, have also reported hypofunction in obese people and there are some experimental findings suggesting that poorer executive function predicts overeating and obesity.^5, 31^

Although we did not formally test for hemispheric specificity, the location of the vmPFC cluster on the right but not on the left is consistent with the observations of impaired decision-making in patients with right-sided vmPFC damage.^33^ Functional imaging studies have implicated the right prefrontal cortex in particular in the regulation of eating behaviour, and overeating has been associated with right frontal lesions (reviewed in Alonso-Alonso and Pascual-Leone^34^). Recent studies exploring the effects of transient transcranial direct current stimulation on eating behaviour have linked the activation of the right prefrontal cortex with decreased craving for foods and increased satiety.^35, 36^ Taken together, there is a convergence of several lines of research implicating the right prefrontal cortex in the control of eating behaviour, and perhaps therefore a prima facie case that the observations here and elsewhere^11^ of cortical thinning in this region are relevant to the behaviours involved in the persistence of elevated BMI. Interestingly, no adiposity-associated cortical thinning was observed in large sample of children (*n*=378), suggesting perhaps that prefrontal thinning, as a potential endophenotype of obesity linked to maladaptive food choices, only exerts its effect in adolescence or adulthood, when children begin making their own food choices.^37^ Altogether, although these reports provide a reasonable fit to our observations, we acknowledge that such a perspective is rather simplistic, based on the assumption of a one-to-one mapping between vmPFC thinning and an alteration in a particular cognitive process. Carefully designed prospective studies would need to be set-up to explore this possibility further.

In comparison with the vmPFC, the finding of cortical thinning in the lateral occipital cortex with increasing BMI was somewhat surprising. Interestingly, a recent study^38^ also found a negative correlation between BMI and thickness of the left LOC, approximately spanning the same region as detected in this study. However, the authors did not provide a specific interpretation of this finding. Equally, obese people have been shown to display a reduced functional connectivity of the left LOC during a picture-viewing task.^39^ This LOC region has been primarily associated with object recognition. It extends from the transverse occipital sulcus/intraparietal sulcus to the middle occipital gyrus and overlaps with regions that have been described as part of the dorsal attention functional network.^40^ This network is thought to direct attention to stimuli based on internal goals and to suppress responses to irrelevant stimuli, which has been described as goal-directed visual attention. Indeed, this same region has been reported to be consistently underactive in executive function tasks in adults and children with attention-deficit hyperactivity disorder.^41^

There are also other strands of research that implicate the occipital cortex/visual attention in obesity. For example, obesity has been associated with reduced functional connectivity of the extrastriate cortex during visual processing of both food and non-food rewards.^42, 43^ Attention bias for food pictures in obesity has been described (reviewed in Nijs and Franken^44^), potentially representing a failure of shifting visual attention from salient food stimuli even after they have been devalued by satiation.^45^ Overall, although the BMI-related thinning of the LOC was not predicted, given that the finding was strong and indeed replicated in another study, we feel that it invites further exploration, particularly with regard to its potential functional implications.

So far, we have focused on the possibility that the structural alterations have a causal role in elevating BMI. But the converse possibility, that the observations are due to elevated BMI, is equally compelling. Indeed, the aforementioned study^37^ that failed to find an association between cortical thickness and overweight in children might also indicate that the BMI-associated thickness changes in the adult sample are secondary to adiposity.

Obesity is associated with several preclinical physiological dysfunctions that affect brain structure. Although our sample of volunteers did not have any clinical manifestations of disease, as we did not conduct laboratory tests of metabolic health, we cannot exclude the presence of subclinical metabolic dysfunctions, such as insulin resistance that could be related to the observed cortical thinning. Metabolically healthy obese individuals with undetected insulin resistance have been reported to have subclinical markers of cardiovascular disease such as increased carotid artery intima media thickness and coronary artery calcium that distinguish them from and confer a higher risk for cardiovascular disease than lean controls.^46^

For example, it is well established that obesity is associated with a state of low-grade systemic inflammation: hypertrophic adipocytes have been linked to the increased secretion of pro-inflammatory cytokines, which in turn have been linked to the disruptions of insulin signalling and insulin resistance.^2^ In rodents, high-fat diets have been shown to induce an inflammatory response and subsequent damage in the hypothalamic region controlling food intake and energy expenditure.^47, 48^ Even though there is no evidence of pro-inflammatory state elsewhere in the brain, systemic markers of inflammation, such as increased levels of interleukin-6, have been linked to decreased global volume,^3^ and to reduced volumes of the hippocampus and the prefrontal cortex.^49^ The fact that calorie restriction abolishes cytokine-driven reduction in brain volume strongly speaks in favour of obesity/inflammation as inducers of brain atrophy.^50^

Leptin has been proposed as another mediator of the effects of obesity on brain structure. Leptin-deficient rodents display several brain structural abnormalities, which can be improved with external leptin administration.^51^ In humans, increased leptin levels in middle age have been associated with a reduced risk of dementia in non-obese people.^52^ On the other hand, in groups of subjects with a wider range of BMIs, elevated levels of leptin have also been linked to brain volume deficits,^53^ perhaps due to leptin resistance.

Obesity has also been linked to increased cortisol secretion,^54^ which in turn has been linked to reduced brain volume.^55^ It is also often associated with increased incidence of hypoxic conditions, such as obstructive sleep apnoea, asthma and several vascular pathologies leading to ischaemia,^56^ all of which have been associated with brain atrophy.^57, 58^

The regional specificity of the thickness effects observed might be viewed as speaking against the adiposity-related insult as the underlying cause of cortical thinning. However, there are certain observations that contest this, suggesting that particular brain regions are indeed more susceptible to atrophy linked to aging or external insults. For instance, it has been reported that the frontal lobe is more susceptible to age-related atrophy,^59^ and that the hippocampus and the prefrontal cortex are more vulnerable to inflammatory damage.^49^ The regional specificity of the observed effects does not by itself rule out the possibility that these changes are consequential to obesity.

We acknowledge several limitations of the study. First, inherent to any cross-sectional design, such as ours, is the inability to determine the timeline of the development of observed differences, and whether the differences are causal or consequential, or if they predispose to obesity and are then worsened by increasing adiposity. Second, we did not have any specific measures of adiposity (such as absolute fat mass) and metabolic health. Third, we did not have any eating behaviour measures that could be related to the observed structural changes.

In summary, we sought to clarify the relationship between BMI and cortical grey matter structure in a large group of adults using structural MRI. Using a surface-based analysis, we were able to systematically examine distinct measures of cortical grey matter. Our findings of regional thinning demonstrate that, even in younger adults with no known clinical morbidities, adiposity is associated with changes in cortical morphology. The observation of vmPFC thinning in particular is highly consistent with the known functions of this region in value-based decision-making and mediating a balance between goal-directed and habitual responding.

## Figures and Tables

**Table 1:** The association of BMI with global measures of cortical grey matter structure

*Dependent variable*	*Covariate*	t*-value*	P-*value*
Cortical thickness	Age, gender, scanner, hemisphere and surface area	−1.18	0.24
Surface area	Age, gender, scanner and hemisphere	0.07	0.95
LGI	Age, gender, scanner, hemisphere and surface area	−0.19	0.85

Abbreviations: BMI, body mass index; LGI, local gyrification index.

**Table 2:** Two clusters (FreeSurfer nomenclature) whose cortical thickness exhibited a negative correlation with BMI

*Region*	*Side*	*Cluster size*		*Peak Talairach coordinates*			*Peak score*	P_*MC*_
		*Vertices*	*mm*^*2*^	x	y	z	t	
Lateraloccipital	L	7504	4825.42	−41	−86	−1	−7.29	0.0001
Medialorbitofrontal	R	6148	3643.09	5	26	−21	−4.39	0.0001

Abbreviations: BMI, body mass index; L, left; R, right.
