# Supplementary material for: Increased body mass index is associated with specific regional alterations in brain structure
Source: Int J Obes (Lond). 2016 Apr 19;40(7):1177–82. doi: 10.1038/ijo.2016.42 (PMC4936515; doi:10.1038/ijo.2016.42)
Supplement: Supplementary Information [file ijo201642x1.docx]

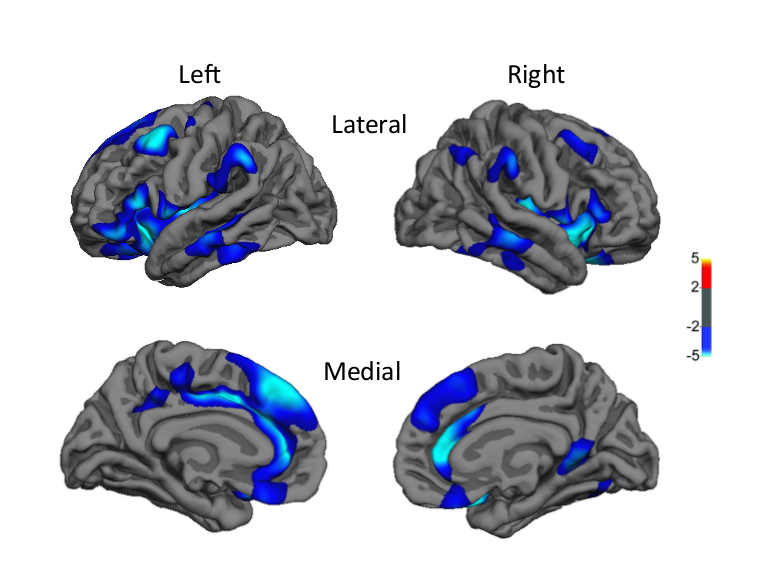


Supplementary Figure 1. Lateral and medial view of the clusters whose cortical thickness exhibited a negative correlation with age. The colour bar represents the logarithmic scale of Monte Carlo cluster-wise corrected p-values (-log(10)p_MC_). Red indicates positive and blue indicates negative association.

Supplementary Table 1**.** Association of obesity with MRI measures of gray matter structure (volume, density, cortical thickness)

|  | Demographics | |  |  |  |  | |  |  |
| --- | --- | --- | --- | --- | --- | --- | --- | --- | --- |
| Study | N | Age±SD | Exclusion criteria | Method | Indepnd. variable | Depnd. variable | Covariates (*and notes) | | Main findings |
| *Cross-sectional studies* | | | | | | | | | |
| Sharkey et al. (1) | 378 (F/M) | Age range: 4 to 18 | Neurological or psychiatric illness, language disorder, substance abuse, prematurity, exposure to toxins in utero | FS (vertex-wise) | BMI | CT | Age, gender | | No significant association between BMI and CT. |
| Veit et al. (2) | 72 (F/M) | 29.65±8.15 | Metabolic, chronic, psychiatric or neurological disease | FS (vertex-wise) | BMI, VAT | CT | Age, gender, total SA, education | | BMI and VAT independently neg. corr. with CT clusters comprising the left lateral occipital area, the left inferior temporal cortex, and the left precentral and inferior parietal area. The right insula, the left fusiform gyrus and the right inferior temporal area only neg. corr. with VAT. |
| Karlsson et al. (3) | 45 (F/M) | 46.5±9.5 (L), 47.3±8.9 (Ob) | Eating disorders, severe mental disorders, alcohol abuse | VBM (voxel-wise) | Ob vs. L | GMV | Total GMV, age, gender, (*some Ob subjects had T2D) | | Ob vs. L: smaller GMV of the right inferior frontal gyrus, inferior temporal gyri bilaterally, right middle temporal gyrus, left postcentral gyrus, occipital gyri bilaterally. |
| Kurth et al. (4) | 115 (F/M) | 45.2±15.5 | Neurological or psychiatric disorders, HypT, T2D, lipid disorders | VBM (voxel-wise) | BMI, WC | GMV | Age, gender | | BMI and WC neg. corr. with GMV of hypothalamus, OFC, frontal pole, inferior and superior frontal gyri, dorsomesial prefrontal cortices, hippocampus, middle and superior temporal gyri, and cerebellum. |
| Marqués-Iturria et al. (5) | 37 (F/M) | 32.3±5.9 (L), 33.7±5.7 (Ob) | Psychiatric or cognitive disorders, motor or sensory disease, MetS | FS (global, vertex-wise) | Ob vs. L | GMV, SA, CT | Age, education, ICV | | Ob vs. L: no difference in any global measure, thinner cortex in the left superior frontal and right medial OFC, and smaller volume of the bilateral diencephalon and brainstem. |
| Rajagopalan et al. (6) | 517 (F/M) | 75.2±7.3 | Not provided | TBM | Leptin conc. | GMV | Age, gender, BMI, insulin  conc. | | Leptin conc. neg. corr. with GMV of frontal, parietal, temporal and occipital lobes, brainstem, and cerebellum. |
| Hassenstab et al. (7) | 53 (F/M) | 48.4±11.3 (SWLM),  47.8±7.6 (Ob),  43.6±8.4 (NOL) | Neurological and psychiatric conditions,  psychiatric and/or weight loss medication | FS (global, ROI-s) | Ob vs. NOL vs. SWLM | GMV, CT | Age, gender, antihypertensive medication | | No group difference for global measures of GMV and CT. Ob vs. NOL (vertex-wise): thinner dorsal anterior cingulate, posterior parietal, and anterior insular cortices bilaterally. |
| Moreno-López et al. (8) | 52(M/F) | 14.1±1.4 (L),  14.2±1.4 (Ov/Ob) | Eating and personality disorders,  medical history | VBM (ROI-s,  voxel-wise) | Ov/Ob vs. L, BMI,  cognitive measures | GMV | Total GMV, gender | | Ov/Ob vs. L: increased GMV of the right hippocampus. Reward sensitivity and UPPS-P positive urgency neg. corr. with GMV of the left secondary somatosensory cortex, Stroop scores pos. corr. with the GMV of the left dlPFC. |
| Mueller et al. (9) | 43 (F/M) | 24.8±3.0 (L), 26.4±5.4 (Ov/Ob) | Cognitive impairment | VBM (voxel-wise) | Serum NSE conc. | GMD | Age, total GMV, serum leptin receptor conc. | | In Ov/Ob subjects, GMD of cerebellum and medial temporal lobe (bilaterally) neg. corr. with NSE conc. |
| Smucny et al. (10) | 53 (F/M) | 31.32±3.45 (OR),  30.29±3.81 (OP) | Eating disorders | VBM (voxel-wise) | OP vs. OR,  leptin conc.,  hunger ratings | GMV | Age, gender,  total GMV | | OP vs. OR: smaller volume of OFC, insula and cerebellum. Serum leptin conc. and hunger ratings neg. corr. with the insula GMV. |
| Cazettes et al. (11) | 63 (F/M) | 57.6±6.7 (L),  58.7±7.7 (Ov/Ob) | Not provided | FS (ROI-s) | Fibrinogen conc. | GMV | Age, HypT, WHR, blood lipids, blood glucose | | In Ov/Ob subjects, fibrinogen conc. neg. corr. with the GMV of the lateral OFC (bilaterally). |
| Horstmann et al. (12) | 122 (F/M) | 25.1±4.4 (F), 25.5±4.3 (M) | HypT, dyslipidaemia, MetS, psychiatric conditions, smoking, T2D | VBM (voxel-wise). | BMI, leptin conc. | GMV | Total GMV, age, gender | | BMI pos. corr. with GMV of the medial posterior OFC, vStr bilaterally, hypothalamus, and the left putamen. Leptin conc. pos. corr. with GMV of the ventral striatum bilaterally and hypothalamus. |
| Debette et al.(13) | 733 (M/F) | 60±9 | Not provided | Custom-made image-analysis software | BMI, WC,  WHR, SAT, VAT | ICV | Age, gender, vascular risk factors, physical activity Index, HOMA-IR, CRP. | | BMI, WC, WHR, VAT, SAT negatively correlated with the ICV. |
| Ho et al. (14) | 206 (F/M) | 76.1 ± 4.7 (*FTO* no-risk genotype),  76.2 ± 5.2 (*FTO* risk genotype) | Cognitive impairment | TBM | *FTO* risk vs. *FTO* no-risk genotype,  BMI. | GMV | Age, gender | | BMI neg. corr. with GMV of frontal, temporal, parietal, and occipital lobe regions. FTO risk allele vs. non-carriers: smaller GMV of frontal lobes and occipital lobes. High BMI and *FTO* risk allele associated with reduced GMV in frontal and occipital lobe regions. |
| Raji et al. (15) | 94 (F/M) | 77.5 ± 4.0 (L),  77.2 ± 2.6 (Ov),  76.9 ± 2.8 (Ob) | Dementia, mild cognitive impairment | TBM | Ob vs. Ob vs. L, BMI | GMV | Insulin conc., T2D, age, gender, ethnicity | | Ob vs. L: lower GMV of the frontal lobes, anterior cingulate gyrus, hippocampus and basal ganglia. Ov vs. L: lower GMV of the basal ganglia, corona radiata and parietal lobe. Ob vs. Ov: no differences. BMI neg. corr. with GMV of the left OFC and anterior cingulate gyrus, bilateral medial temporal lobe. |
| Walther et al. (16) | 95 (F) | 71.0 ± 9.8 (L),  69.9 ± 8.1 (Ov),  66.9 ± 9.9 (Ob) | History of head injury, neurological or psychiatric disorder, drug and/or alcohol abuse | VBM  (voxel-wise) | BMI | GMV | ICV, age, HypT | | BMI neg. corr. with GMV of the left orbitofrontal gyrus, right inferior and precentral frontal cortex, right posterior cortex, and right posterior and lateral cerebellum. |
| Gunstad et al. (17) | 209 (F/M) | 33±14.6 (L),  42.2±16.5 (Ov),  45.1±15.3 (Ob) | Traumatic brain injury, neurological disorder, HypT, T2D, cardiac disease, thyroid disease | VBM  (global, ROI-s) | L vs. Ov vs. Ob, BMI. | GMV, ICV | Age | | Ob vs. L/Ov: smaller ICV and GMV. BMI neg. corr. with GMV of parietal and temporal lobes. |
| Taki et al. (18) | 1428 (F/M) | 46.4±14.1 (F), 44.5 ± 16.1 (M) | Central nervous system disease, brain injury | VBM (global, vertex-wise) | BMI | GMR, GMV | Age, gender,  alcohol intake, HypT, T2D | | Men: BMI neg. corr. with global GMR, women: no association between BMI and GMR. In men, BMI neg. corr. with GMV of the bilateral temporal, cerebellum, bilateral fusiform gyrus, bilateral frontal lobes, bilateral precuneus, and midbrain, BMI pos. corr. with GMV of the bilateral inferior frontal gyri, cerebellum, bilateral frontal and temporal lobes, thalamus and caudate. In women, no association between BMI and GMV. |
| Pannacciulli et al. (19) | 60 (F/M) | 32±8 (L),  33±9 (Ob) | Alcohol and drug abuse, endocrine disorders, HypT, pulmonary, cardiovascular, gastrointestinal, hepatic, renal, and central nervous system disorders | VBM (voxel-wise) | Ob vs. L, BMI, blood glucose, insulin conc. | GMD | Age, gender, handnesn-esses, global GMD | | Ob vs. L: lower GMD in the right cerebellum, left post-central gyrus, right frontal operculum, right and left putamen, and right and left middle frontal gyri, equally, higher GMD in the left calcarine cortex, left middle occipital gyrus, left inferior frontal gyrus, and right cuneus. In Ob subjects, BMI neg. corr. with GMD of the left post-central gyrus. No associations of GMD with glucose or insulin conc. |
| Ward et al. (19) | 117 (F/M) | 54.2±6.5 | Axis I psychiatric disease, history of major medical conditions, cognitive impairment | VBM | BMI | NBV | Age, gender, family history of AD, APOE genotype,  total cholesterol, BP. | | BMI neg. corr. with NBV. |
| *Longitudinal studies* | | | | | | | | | |
| Bobb et al. (20) | 347 (M) | 61.5±8 (L),  60±8.2 (Ov),  59.8±7.3 (Ob) | Not provided | VBM (global, ROI-s, voxel-wise) | BMI | GMV | Age, ethnicity, ICV, smoking, APOE genotype | | BMI neg. corr. with ICV, total GMV, GMV-s of frontal, temporal, parietal, occipital lobes, insula, amygdala, cingulate gyrus. Higher baseline BMI predicts greater decline in temporal and occipital GM ROI volumes over the 5-year period. |
| Yokum et al. (21) | 83 (F),  recruited as part of studies S1 and S2 | 15.7±0.9 (S1),  20.7±1.5 (S2) | Psychiatric disorder, use of psychoactive drugs | VBM (ROI-s, voxel-wise) | Ob vs. Ov vs. L, BMI | GMV,  WMV | Total GMV and WMV | | Ob vs. Ov/L: reduced total GMV. L vs. Ov: no difference in total GMV. Ob vs. Ov: reduced total WMV. Ov/Ov vs. L: no difference in total WMV. BMI pos. corr. with GMV of the right middle occipital gyrus. Reduced GMV in bilateral superior frontal gyrus predicts increase in BMI at 1-year follow-up (trend-level). |

Blood pressure (BP), C-reactive protein (CR), cortical thickness (CT), Gray matter density (GMD), Gray matter ratio = ratio of gray matter volume and intracranial volume (GMR), Gray matter volume (GMV), homeostasis model assessment of insulin resistance (HOMA-IR), hypertension (HypT), Intracranial volume (ICV), lean (L), metabolic syndrome (MetS), Normalised brain volume = ratio of the total brain volume (gray matter + white matter) and intracranial volume (NBV), never-obese lean (NOL), neuron-specific enolase (NSE), obese (Ob), obesity-prone (OP), obesity-resistant (OR), overweight (Ov), surface area (SA), subcutaneous adipose tissue (SAT), successful weight loss maintainers (SWLM), tensor-based morphometry (TBM), type 2 diabetes (T2D), visceral adipose tissue (VAT), waist circumference (WC), waist-to-hip ratio (WHR), white matter volume (WM

Supplementary table 2. Clusters (FreeSurfer nomenclature) whose cortical thickness exhibited a negative correlation with age

|  |  | Cluster size | | Peak MNI coordinates (FreeSurfer) | | | Peak score |  |
| --- | --- | --- | --- | --- | --- | --- | --- | --- |
| Region | Side | Vertices | mm^2^ | x | y | z | T | p(MC) |
| Lateralorbitofrontal | L | 31574 | 14853.26 | -26.2 | 22.4 | -10.1 | -7.06 | 0.0001 |
| Insula | R | 14866 | 6460.91 | 29.3 | 17.5 | -7.3 | -6.96 | 0.0001 |
| Caudalmiddlefrontal | L | 4059 | 2124.91 | -41 | 13.5 | 48.7 | -5.84 | 0.0001 |
| Middletemporal | L | 3804 | 2128.39 | -55 | -25 | -13.6 | -4.61 | 0.0001 |
| Middletemporal | R | 4072 | 2232.45 | 61.9 | -27.5 | -11.7 | -4.47 | 0.0001 |
| Isthmuscingulate | R | 2537 | 1040.82 | 15.2 | -47.5 | 2.1 | -4.27 | 0.0067 |
| Supramarginal | R | 1795 | 793.77 | 60.2 | -27.5 | 37.7 | -3.96 | 0.0283 |
| Superiorfrontal | R | 2097 | 1125.45 | 7.6 | 49 | 30.2 | -3.42 | 0.038 |
| Caudalmiddlefrontal | R | 1194 | 784.64 | 38.8 | 15 | 51.5 | -3.39 | 0.0298 |
| Inferiorparietal | R | 2003 | 885.77 | 47.9 | -56.3 | 45.2 | -3.18 | 0.0165 |
| Fusiform | R | 1141 | 747.05 | 28.8 | -71 | -7.5 | -2.47 | 0.0387 |

**References**

1. Sharkey RJ, Karama S, Dagher A. Overweight is not associated with cortical thickness alterations in children. Front Neurosci [Internet]. 2015 Feb 4 [cited 2015 Dec 14];9. Available from: http://www.ncbi.nlm.nih.gov/pmc/articles/PMC4316697/

2. Veit R, Kullmann S, Heni M, Machann J, Häring H-U, Fritsche A, et al. Reduced cortical thickness associated with visceral fat and BMI. NeuroImage Clin. 2014;6:307–11.

3. Karlsson HK, Tuulari JJ, Hirvonen J, Lepomäki V, Parkkola R, Hiltunen J, et al. Obesity is associated with white matter atrophy: a combined diffusion tensor imaging and voxel-based morphometric study. Obes Silver Spring Md. 2013 Dec;21(12):2530–7.

4. Kurth F, Levitt JG, Phillips OR, Luders E, Woods RP, Mazziotta JC, et al. Relationships between gray matter, body mass index, and waist circumference in healthy adults. Hum Brain Mapp. 2013 Jul;34(7):1737–46.

5. Marqués-Iturria I, Pueyo R, Garolera M, Segura B, Junqué C, García-García I, et al. Frontal cortical thinning and subcortical volume reductions in early adulthood obesity. Psychiatry Res. 2013 Nov 30;214(2):109–15.

6. Rajagopalan P, Toga AW, Jack CR, Weiner MW, Thompson PM, Alzheimer’s Disease Neuroimaging Initiative. Fat-mass-related hormone, plasma leptin, predicts brain volumes in the elderly. Neuroreport. 2013 Jan 23;24(2):58–62.

7. Hassenstab JJ, Sweet LH, Del Parigi A, McCaffery JM, Haley AP, Demos KE, et al. Cortical thickness of the cognitive control network in obesity and successful weight loss maintenance: a preliminary MRI study. Psychiatry Res. 2012 Apr 30;202(1):77–9.

8. Moreno-López L, Soriano-Mas C, Delgado-Rico E, Rio-Valle JS, Verdejo-García A. Brain Structural Correlates of Reward Sensitivity and Impulsivity in Adolescents with Normal and Excess Weight. PLOS ONE. 2012 Nov 21;7(11):e49185.

9. Mueller K, Sacher J, Arelin K, Holiga S, Kratzsch J, Villringer A, et al. Overweight and obesity are associated with neuronal injury in the human cerebellum and hippocampus in young adults: a combined MRI, serum marker and gene expression study. Transl Psychiatry. 2012;2:e200.

10. Smucny J, Cornier M-A, Eichman LC, Thomas EA, Bechtell JL, Tregellas JR. Brain structure predicts risk for obesity. Appetite. 2012 Dec;59(3):859–65.

11. Cazettes F, Cohen JI, Yau PL, Talbot H, Convit A. Obesity-mediated inflammation may damage the brain circuit that regulates food intake. Brain Res. 2011 Feb 10;1373:101–9.

12. Horstmann A, Busse FP, Mathar D, Müller K, Lepsien J, Schlögl H, et al. Obesity-Related Differences between Women and Men in Brain Structure and Goal-Directed Behavior. Front Hum Neurosci. 2011;5:58.

13. Debette S, Beiser A, Hoffmann U, Decarli C, O’Donnell CJ, Massaro JM, et al. Visceral fat is associated with lower brain volume in healthy middle-aged adults. Ann Neurol. 2010 Aug;68(2):136–44.

14. Ho AJ, Stein JL, Hua X, Lee S, Hibar DP, Leow AD, et al. A commonly carried allele of the obesity-related FTO gene is associated with reduced brain volume in the healthy elderly. Proc Natl Acad Sci. 2010 Apr 5;107(18):8404–9.

15. Raji CA, Ho AJ, Parikshak NN, Becker JT, Lopez OL, Kuller LH, et al. Brain structure and obesity. Hum Brain Mapp. 2010 Mar;31(3):353–64.

16. Walther K, Birdsill AC, Glisky EL, Ryan L. Structural brain differences and cognitive functioning related to body mass index in older females. Hum Brain Mapp. 2010 Jul 1;31(7):1052–64.

17. Gunstad J, Paul RH, Cohen RA, Tate DF, Spitznagel MB, Grieve S, et al. Relationship between body mass index and brain volume in healthy adults. Int J Neurosci. 2008 Nov;118(11):1582–93.

18. Taki Y, Kinomura S, Sato K, Inoue K, Goto R, Okada K, et al. Relationship between body mass index and gray matter volume in 1,428 healthy individuals. Obes Silver Spring Md. 2008 Jan;16(1):119–24.

19. Ward MA, Carlsson CM, Trivedi MA, Sager MA, Johnson SC. The effect of body mass index on global brain volume in middle-aged adults: a cross sectional study. BMC Neurol. 2005 Dec 2;5:23.

20. Bobb JF, Schwartz BS, Davatzikos C, Caffo B. Cross-sectional and longitudinal association of body mass index and brain volume. Hum Brain Mapp. 2014 Jan 1;35(1):75–88.

21. Yokum S, Ng J, Stice E. Relation of regional gray and white matter volumes to current BMI and future increases in BMI: a prospective MRI study. Int J Obes. 2012 May;36(5):656–64.
